# CD4 T-cell transcriptome analysis reveals aberrant regulation of STAT3 and Wnt signaling pathways in rheumatoid arthritis: evidence from a case–control study

**DOI:** 10.1186/s13075-015-0590-9

**Published:** 2015-03-22

**Authors:** Hua Ye, Jing Zhang, Jun Wang, Yanyan Gao, Yan Du, Chun Li, Minghua Deng, Jianping Guo, Zhanguo Li

**Affiliations:** Department of Rheumatology and Immunology, Peking University People’s Hospital, 11 South Xizhimen Street, Beijing, 100044 China; School of Mathematical Sciences, Center for Quantitative Biology, Peking University, 136 North Zhong-guan-cun Street, Beijing, 100871 China

## Abstract

**Introduction:**

Rheumatoid arthritis (RA) is a systemic autoimmune disease in which T cells play a pivotal role in the pathogenesis. Knowledge in terms of the CD4 T-cell transcriptome in RA is limited. The aim of this study was to examine the whole-genome transcription profile of CD4 T cells in RA by comparing patients with RA to healthy controls.

**Methods:**

Peripheral blood CD4 T cells were isolated from 53 RA patients with active disease and 45 healthy individuals; 13 cases and 10 controls were enrolled in microarray analysis. The remaining 40 cases and 35 controls were recruited as an independent cohort for the validation study. Bioinformatics was performed on Gene Ontology (GO), gene-gene interaction networks, and pathway analysis. The gene modules, by combining the results from GO, gene networks, and pathway analysis, were selected for further validation.

**Results:**

The CD4 T cells showed 1,496 differentially expressed (DE) genes in RA patients relative to healthy individuals. GO analysis revealed that the DE genes were enriched in immune response, T-cell response, apoptosis process, and Wnt receptor signaling. Pathway analysis also identified that ‘Wnt signaling pathway’ was differentially regulated between two groups (*P* = 2.78 × 10^−10^). By gene-gene network analysis, we found that the DE genes were enriched in T-cell receptor (TCR), JAK-STAT signaling, and Wnt signaling pathway. By gene module analysis, we found that a number of DE genes overlapped in the three different analyses. In total, 23 genes were selected for further validation, and nine genes were confirmed. Of these, four genes (SOCS3, CBL, IFNAR1, and PIK3CA) were involved in STAT3 (signal transducer and activator of transcription 3) signaling, and three genes (CBL, KLF9, and CSNK2A1) were involved in the Wnt signaling pathway. Additionally, several zinc finger transcription factors (ZEB1, ZNF292, and ZNF644) were confirmed.

**Conclusions:**

We report here the first case–control study of the CD4 T-cell transcriptome profile in RA. Our data provide evidence that CD4 T cells from patients with RA have abnormal functional networks in STAT3 signaling and Wnt signaling. Our results also suggest that the aberrant expression of several zinc finger transcription factors (ZEB1, ZNF292, and ZNF644) may be potential pathogenic factors for RA.

**Electronic supplementary material:**

The online version of this article (doi:10.1186/s13075-015-0590-9) contains supplementary material, which is available to authorized users.

## Introduction

Rheumatoid arthritis (RA) is a chronic and systemic autoimmune disease characterized by symmetric inflammation in peripheral joints, leading to cartilage destruction and bone erosion. The etiology of RA remains elusive, but it is believed that genetic factors play an important role in RA pathogenesis. Multiple genes contribute to disease susceptibility and heterogeneous clinical manifestations [1,2]. To gain insight into the molecular signature underlying disease pathogenesis, gene expression profiling studies have emerged as a powerful way to comprehensively identify the genes that are differentially expressed in blood and tissues between patients and healthy individuals [3].

To date, a number of transcriptome studies have focused on peripheral blood mononuclear cells (PBMCs) or fibroblast-like synoviocytes (FLSs) to understand the aberrant biological pathways involved in the pathogenesis of RA [4-9]. Yet although much has been learned about T cells in RA pathology, knowledge is limited in terms of whole-genome transcription profiling of CD4 T cells in RA. One microarray study was conducted on CD4 T cells in RA but with a case-only design [10]. To better understand the complex molecular mechanisms and discover the potential predictive biomarkers for RA, we performed a case–control study of CD4 T-cell transcriptome analysis by comparing RA patients to healthy controls. We found a great difference in gene expression profiling of CD4 T cells between active RA cases and healthy controls and discovered several aberrant signaling pathways in CD4 T cells from patients with RA. Finally, by quantitative real-time polymerase chain reaction (qPCR) validation, we identified nine genes involved in STAT3 (signal transducer and activator of transcription 3) signaling, Wnt signaling pathway, and zinc finger transcription regulation.

## Methods

### Study subjects

Thirteen patients with RA and nine healthy controls were enrolled in microarray analysis. Forty RA cases and 35 healthy individuals were subsequently recruited for the validation study. All patients satisfied the American College of Rheumatology 1987 revised criteria for a diagnosis of RA [11] and were recruited between March 2009 and September 2011. All cases had active disease at the time of blood sampling: Disease Activity Score (DAS) 28 of 5.55 (range of 4.07 to 8.26). The patients maintained the same doses of methotrexate no less than 3 months and did not concurrently receive other disease-modifying anti-rheumatic drugs or biologics before the study. The baseline demographic characteristics of patients and healthy controls are detailed in Table S1 (Additional file 1). The study was approved by the Medical Ethics Committee of Peking University People’s Hospital, and informed consent was obtained from all participants.

### CD4^+^ T-cell RNA processing

In total, 12-mL whole blood samples were drawn and stored at 4°C for less than 4 hours. Samples were layered onto Ficoll-Paque Plus, and PBMCs were separated by density gradient centrifugation. An automated magnetic beadbased-positive selection protocol was used to isolate CD4 cells (Stemcell Technologies, Vancouver, BC, Canada). By this approach, a median purity of 95% CD4^+^ T cells was achieved by flow cytometry analysis. Total CD4^+^ T-cell RNA was extracted by using TRIzol reagent (Invitrogen, Carlsbad, CA, USA) and purified by using an RNeasy Mini Kit (Qiagen, Hilden, Germany). Only RNA preparations with a 28S/18S ratio of more than 1.7 and an A260/280 range of 1.8 to approximately 2.1 were used for gene expression analysis.

### Microarray assay

The transcriptome analyses used Human Genome U133 Plus version 2.0 high-density oligonucleotide arrays (Affymetrix, Santa Clara, CA, USA) with 54,000 probe sets and 1,300,000 distinct oligonucleotides to interrogate 47,000 well-characterized human transcripts. The sample labeling, microarray hybridization, and washing were performed on the basis of the standard protocols of the manufacturer. Briefly, total RNA was transcribed to double-strand cDNA and then to synthesized cRNA and labeled with cyanine-3-CTP. The labeled cRNAs were hybridized onto the microarray. After the slides were washed, the arrays were scanned by GeneChip Scanner 3000 (Affymetrix). The gene expression array data were digitalized by using GeneChip Operating Software (version 1.4; Affymetrix) and normalized by eliminating the highest and lowest 2% of the data by using MAS5 algorithm (Affymetrix). The microarray data have been deposited in the Gene Expression Omnibus (GEO) of the National Center for Biotechnology Information (NCBI) and are accessible through GEO series accession number GSE56649.

### Real-time quantitative polymerase chain reaction

The selected candidate genes were validated by qPCR. Briefly, the cDNA was synthesized in accordance with the instructions indicated in a RevertAid™ First-Strand cDNA Synthesis Kit (Fermentas, Shenzhen, China). Two-step PCR was performed by using SYBR Green PCR Master Mix (Applied Biosystems, Foster City, CA, USA) in accordance with the instructions of the manufacturer. The reaction was run on an ABI 9700 fluorescent sequence detection system (Applied Biosystems). Gene expression was quantified relative to the expression of the housekeeping gene 18 s rRNA and normalized to control by standard 2^−△△CT^ calculation. Primer sequences used are summarized in Table S2 (Additional file 2).

### Statistical analysis

For microarray analysis, a differentially expressed (DE) gene was defined if its geometric mean of intensities reached at least 1.5-fold changes between case and control groups. The false-discovery rate (FDR) was applied to determine the statistical significance. An FDR-adjusted *P* value (*q* value) of below 0.05 was defined as statistically significant. The independent-samples *t* test was applied for the analysis of candidate gene validation, and a *P* value of less than 0.05 was considered statistically significant after Bonferroni correction. All statistical analyses were conducted by using program SPSS 13.0 (SPSS Inc., Chicago, IL, USA).

### Gene Ontology, gene-gene network, and pathway analysis

The significant DE genes from microarray were first analyzed for hierarchical clustering (Cluster 3.0, available at [12]) and visualized with Treeview 3.0 (available at [13]). To interpret biological meaning of the transcripts, the DE genes were functionally categorized according to the Gene Ontology (GO) database [14]. GO analysis allowed us to classify the large gene list into functionally related gene sets according to a reference (NCBI: *Homo sapiens* genes). Fisher’s exact test and chi-squared test were used to statistically classify GO categories, and an FDR (*q* value) of less than 0.05 was considered statistically significant. The gene-gene interaction networks and pathway analysis were analyzed by using Ingenuity Pathway Analysis software version 7.5 [15]. Interactions among the DE genes were investigated according to the Human Protein Reference Database (HPRD) [16] and the Molecular INTeraction (MINT) database [17]. The significance of the interactions was presented as gene modules and evaluated according to between-ness centrality reflecting the importance of a module in relation to other modules [18-20]. Pathway analysis was applied to find out the significant pathways among DE genes according to the Kyoto Encyclopedia of Genes and Genomes (KEGG) [21]. Pathways generated by fewer than five uploaded genes were excluded from the analysis. Pathway enrichment analysis (PEA) (those with the highest percentage of genes in a particular pathway) was applied to evaluate the significance of pathways [22-24].

## Results

### CD4 T-cell transcriptome in patients with active rheumatoid arthritis versus healthy controls

**Figure 1:**
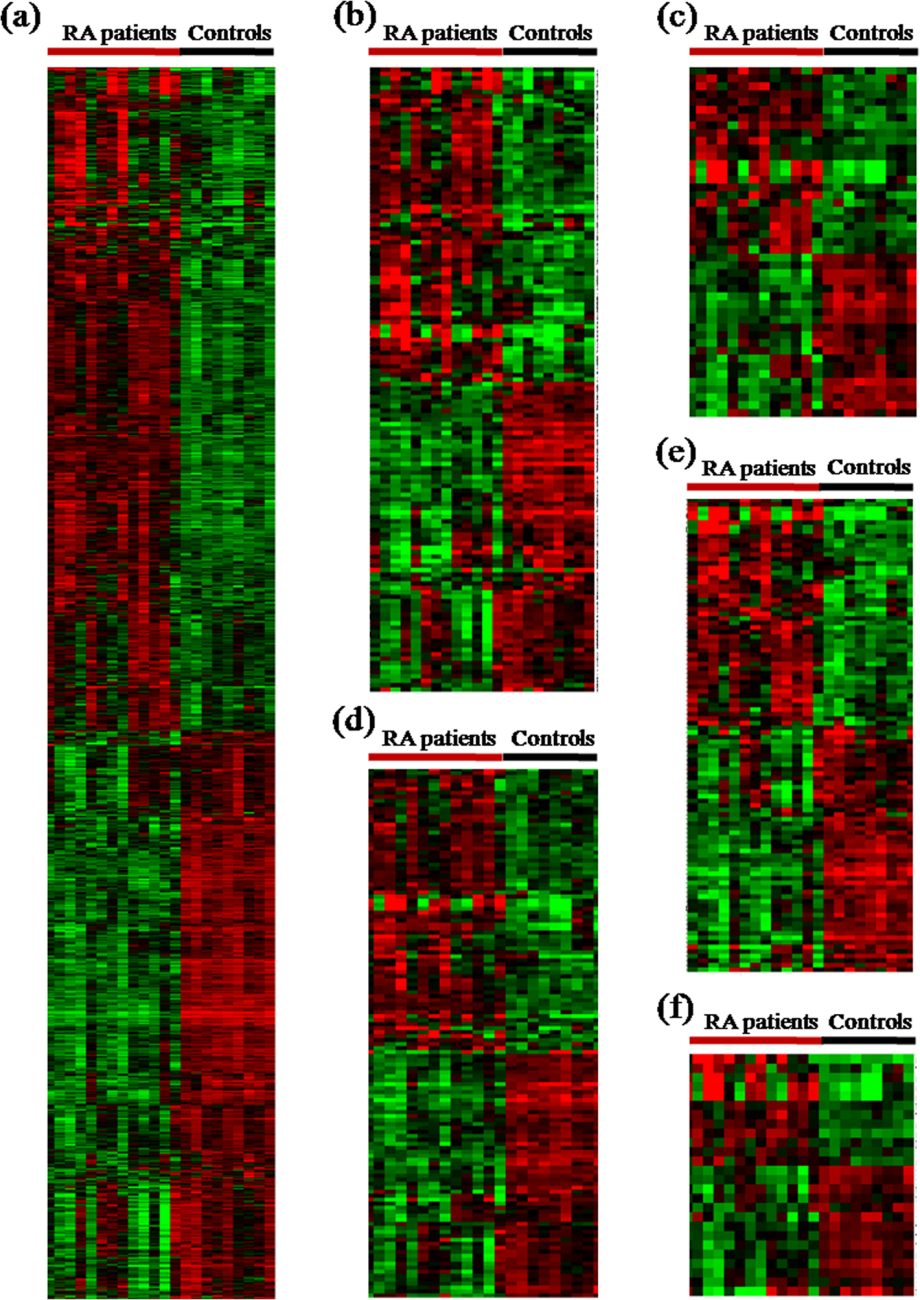
**Cluster diagram of all differentially expressed genes and those classified according to Gene Ontology analysis.** Genes are organized by hierarchical clustering based on overall similarity in expression patterns. Red represents relative expression greater than the median expression level across all samples, and green represents an expression level lower than the median. Black indicates intermediate expression. **(a)** Visualization of 1,495 transcripts able to distinguish active rheumatoid arthritis (RA) from healthy controls in CD4 T cells. **(b)** Visualization of 122 transcripts able to distinguish active RA from controls in immune response. **(c)** Visualization of 40 transcripts able to distinguish active RA from controls in T-cell response. **(d)** Visualization of 111 transcripts able to distinguish active RA from controls in apoptosis process. **(e)** Visualization of 89 transcripts able to distinguish active RA from controls in regulation of kinase and phosphatase activity. **(f)** Visualization of 23 transcripts able to distinguish active RA from controls in regulation of Wnt receptor signaling.

The transcriptome profiles of CD4 T cells from 13 cases with active RA and nine healthy controls were accessed by microarrays. Using a *q* value of less than 0.05 and taking into account genes with at least 1.5-fold changes, we identified a total of 1,496 DE transcripts. The proportion of DE transcripts was substantially higher among RefSeq genes (89%) than non-RefSeq genes (11%). Among the transcripts, 832 were upregulated (Table S3 in Additional file 3) and the remaining 664 were downregulated (Table S4 in Additional file 4). These genes clearly separated patients with active RA from healthy individuals and were visualized in a hierarchical clustering diagram (Figure 1a).

### Gene Ontology analysis of differentially expressed transcripts defined five major gene categories

By GO analysis, the common genes can be generally classified into three GO systems: biological process, cellular component and molecular function, and (more specifically) ‘higher level’ descriptive GO terms (for example, ‘signal transduction’ or ‘regulation of apoptosis’). We found that the DE genes in CD4 T cells were enriched in several common ontologies, including immune response (GO:0006955, 122 genes, *q =* 5.99 × 10^−8^), more specifically in (i) regulation of adaptive immune response (GO:0002819); (ii) positive regulation of chronic inflammatory response (GO:0002678); (iii) interferon-gamma (IFNγ)-mediated signaling pathway (GO:0060333); and (iv) response to cytokine stimulus (GO:0034097), apoptosis process (111 genes, *q =* 1.73 × 10^−5^), more specifically in (i) induction of apoptosis by intracellular signals (GO:0008629); (ii) cellular component involved in apoptosis (GO:0006921); (iii) regulation of myeloid cell apoptosis (GO:0033032); and (iv) recognition of apoptotic cell (GO:0043654), T-cell receptor (TCR) signaling pathway (GO:0050852, 40 genes, *q =* 2.98 × 10^−6^), and regulation of kinase (GO:0007243, 58 genes, *q =* 3.29 × 10^−4^) and phosphatase (GO:0016310, 31 genes, *q =* 9.36 × 10^−4^) activity (Figure 1b-e, Table S5-8 in Additional files 5, 6, 7 and 8). Interestingly, of the significantly enriched GO categories, one was related to the Wnt receptor signaling pathway (GO:0016055, 23 genes, *q =* 4.15 × 10^−4^) (Figure 1f, Table S9 in Additional file 9). A similar result was obtained by pathway analysis, which identified ‘Wnt signaling pathway’ with great significance of differential regulation between two groups (*q* = 2.78 × 10^−10^). The five major GO terms are summarized in Table 1.

**Table 1:** **Gene Ontology in differentially expressed genes**

**GO_Id**	**GO_term**	**Count**	***Q*** **value**
GO:0006955	Immune response	122	5.99 × 10^−8^
GO:0002819	Regulation of adaptive immune response	23	9.37 × 10^−4^
GO:0002678	Positive regulation of chronic inflammatory response	12	1.38 × 10^−3^
GO:0060333	Interferon-gamma-mediated signaling pathway	9	6.02 × 10^−3^
GO:0034097	Response to cytokine stimulus	17	8.61 × 10^−3^
GO:0006915	Apoptosis	111	1.73 × 10^−5^
GO:0008629	Induction of apoptosis by intracellular signals	17	4.15 × 10^−4^
GO:0006921	Cellular component involved in apoptosis	29	2.38 × 10^−3^
GO:0033032	Regulation of myeloid cell apoptosis	22	7.18 × 10^−3^
GO:0043654	Recognition of apoptotic cell	7	8.42 × 10^−2^
GO:0050852	T-cell receptor signaling pathway	40	2.98 × 10^−6^
GO:0007243	Intracellular protein kinase cascade	58	3.29 × 10^−4^
GO:0016310	Phosphorylation	31	9.36 × 10^−4^
GO:0016055	Wnt receptor signaling pathway	23	4.15 × 10^−4^

### Corresponding interaction networks among differentially expressed genes in the five major Gene Ontology categories

To identify more significant DE genes, the network interactions among DE genes implicated in the five major GO categories were investigated according to HPRD and MINT databases. As shown in Table S10 (Additional file 10), the DE gene modules, such as PIK3R1, PAK1, CBL, PIK3CA, SYK, and ZAP70, were highly relevant to TCR signaling and JAK-STAT signaling. A cluster of genes involved in Wnt signaling (for example, TLE1, CSNK2A1, and MAPK8) were also present in this network. Additionally, several gene modules with possible functional involvement in RA pathogenesis, such as ZAP70, HSP90AA1, HSP90AB1, and VIM, were depicted in the network.

### Corresponding signaling pathways of differentially expressed genes

**Figure 2:**
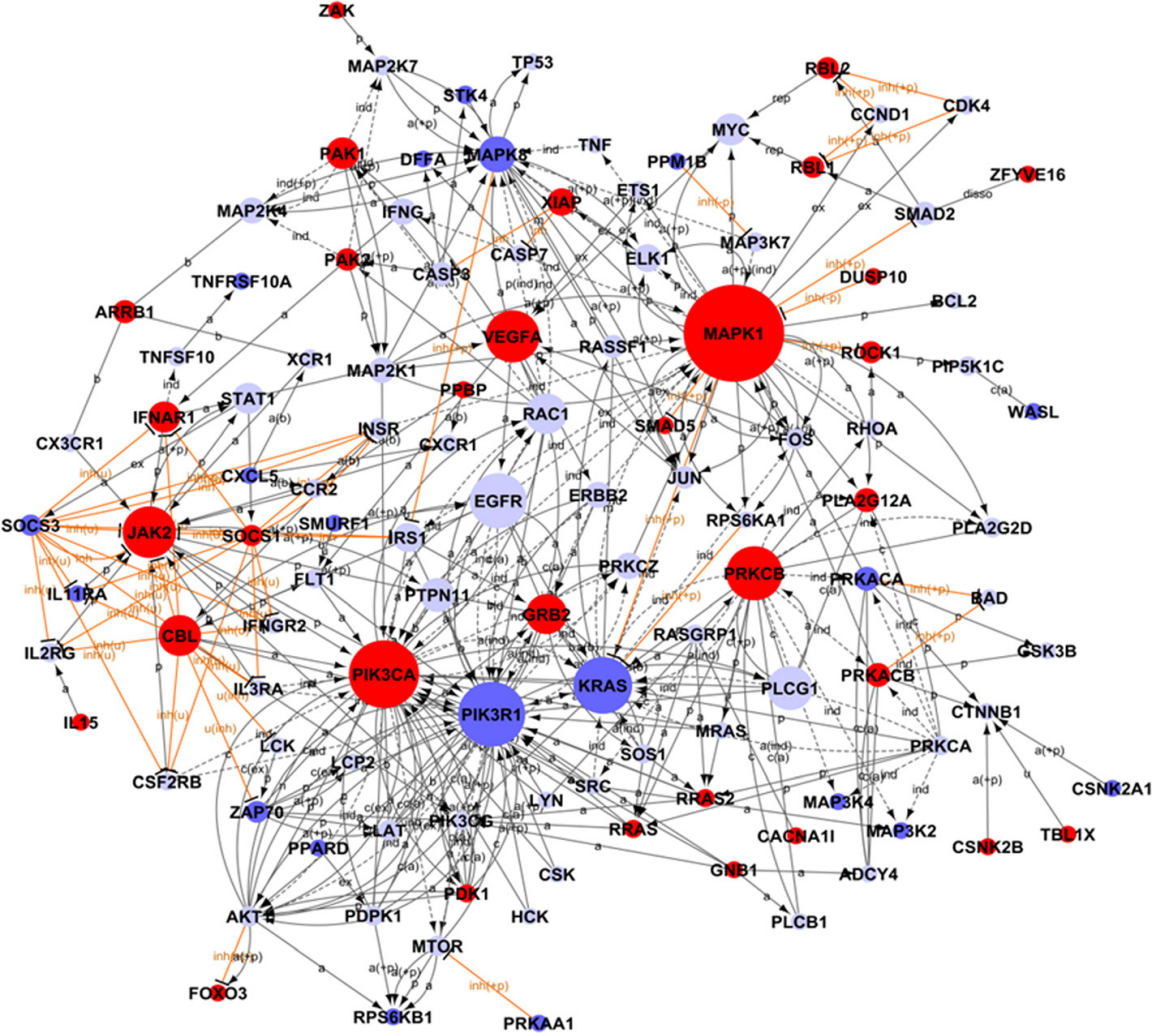
**Network representations of the differentially expressed genes presented in pathway analysis.** Genes are represented as individual nodes. The biologic relation between two nodes is represented as an edge (line). The node size indicates the importance of a gene module in relation to other modules. The color of the node indicates upregulated (red) or downregulated (green) genes.

The possible signaling pathways implicated in regulation of CD4 T cells in active RA were investigated according to the KEGG database. The high-ranked pathways are displayed in Table 2. The DE genes were highly relevant to T-cell response, such as the ErbB signaling pathway (PEA *=* 28.122, *q* = 4.88 × 10^−14^), the TCR signaling pathway (PEA *=* 24.542, *q* = 2.69 × 10^−14^), the mammalian target of rapamycin (mTOR) signaling pathway (PEA *=* 23.525, *q* = 1.71 × 10^−7^), and the TGF-β signaling pathway (PEA *=* 23.435, *q* = 2.63 × 10^−11^). In addition, several canonical pathways were depicted, such as apoptosis (PEA *=* 22.909, *q* = 3.01 × 10^−11^), the chemokine signaling pathway (PEA *=* 19.114, *q* = 1.52 × 10^−17^), the MAPK signaling pathway (PEA *=* 17.304, *P* = 2.14 × 10^−21^), and the Jak-STAT signaling pathway (PEA *=* 15.785, *q* = 2.44 × 10^−11^). Interestingly, the Wnt signaling pathway (PEA *=* 14.755, *q* = 2.78 × 10^−10^) and the systemic lupus erythematosus (SLE) signaling pathway (PEA *=* 7.282, *q* = 3.47 × 10^−4^) were also depicted in this analysis. Furthermore, by pathway network analysis, several genes, such as MAPK1, PIK3CA, PIK3R1, KRAS, and PRKCB, were centered in the network (Figure 2). The majority of these genes overlapped with the ones in the interaction network analysis (Table S11, Additional file 11).

**Table 2:** **Identification of signaling pathways based on Kyoto Encyclopedia of Genes and Genomes in differentially expressed genes**

**Path_Id**	**Path_name**	**Path_diffgenes**	**Path_genes**	**Enrichment**	***Q*** **value**
4012	ErbB signaling pathway	12	87	28.122	4.88 × 10^−14^
4660	T-cell receptor signaling pathway	13	108	24.542	2.69 × 10^−14^
4150	mTOR signaling pathway	6	52	23.525	1.71 × 10^−7^
4350	TGF-β signaling pathway	10	87	23.435	2.63 × 10^−11^
4210	Apoptosis	10	89	22.909	3.01 × 10^−11^
4062	Chemokine signaling pathway	18	192	19.114	1.52 × 10^−17^
4010	MAPK signaling pathway	23	271	17.304	2.14 × 10^−21^
4630	Jak-STAT signaling pathway	12	155	15.785	2.44 × 10^−11^
4310	Wnt signaling pathway	11	152	14.755	2.78 × 10^−10^
4620	Toll-like receptor signaling pathway	5	101	10.093	9.23 × 10^−5^
4060	Cytokine-cytokine receptor interaction	12	263	9.303	6.59 × 10^−9^
5322	Systemic lupus erythematosus	5	140	7.282	3.47 × 10^−4^

Finally, we performed gene module analysis to combine the results from GO, gene-gene interaction, and pathway analysis and found that a substantial number of genes in the three different analyses overlapped. In total, 23 genes were selected for further validation using a cut-off *q* value of 0.05 and an exclusion of being extensively studied in RA according to publications. These genes are characterized in Table 3.

**Table 3:** **Gene modules resulting from the combination of Gene Ontology, gene-gene interaction, and pathway analysis**

**Probe set ID**	**Gene symbol**	**Fold change**	***Q*** **value**
229010_at	*CBL*	2.22	0
212435_at	*TRIM33*	0.61	0
226294_x_at	*FAM91A1*	0.33	0
227817_at	*PRKCB*	2.26	6.101 × 10^−16^
227073_at	*MAP3K2*	0.50	1.296 × 10^−14^
214578_s_at	*ROCK1*	2.99	4.303 × 10^−12^
212073_at	*CSNK2A1*	0.32	5.813 × 10^−8^
1555613_a_at	*ZAP70*	0.59	6.82 × 10^−6^
223300_s_at	*CCDC82*	0.51	6.82 × 10^−6^
212366_at	*ZNF292*	0.59	2.31 × 10^−5^
214917_at	*PRKAA1*	0.57	7.15 × 10^−5^
212249_at	*PIK3R1*	0.58	0.000141
231777_at	*CSNK2B*	3.90	0.00076
37152_at	*PPARD*	0.44	0.000927
231798_at	*NOG*	1.62	0.00133
225661_at	*IFNAR1*	2.16	0.002
235980_at	*PIK3CA*	1.70	0.00264
206359_at	*SOCS3*	0.62	0.00336
226660_at	*RPS6KB1*	0.61	0.0101
212764_at	*ZEB1*	1.83	0.0125
1553725_s_at	*ZNF644*	0.59	0.0187
203542_s_at	*KLF9*	0.61	0.0238
211930_at	*HNRNPA3*	0.64	0.0344

### Validation analysis confirmed that STAT3 profiling and Wnt signaling are the prominent active rheumatoid arthritis ‘signature’

**Figure 3:**
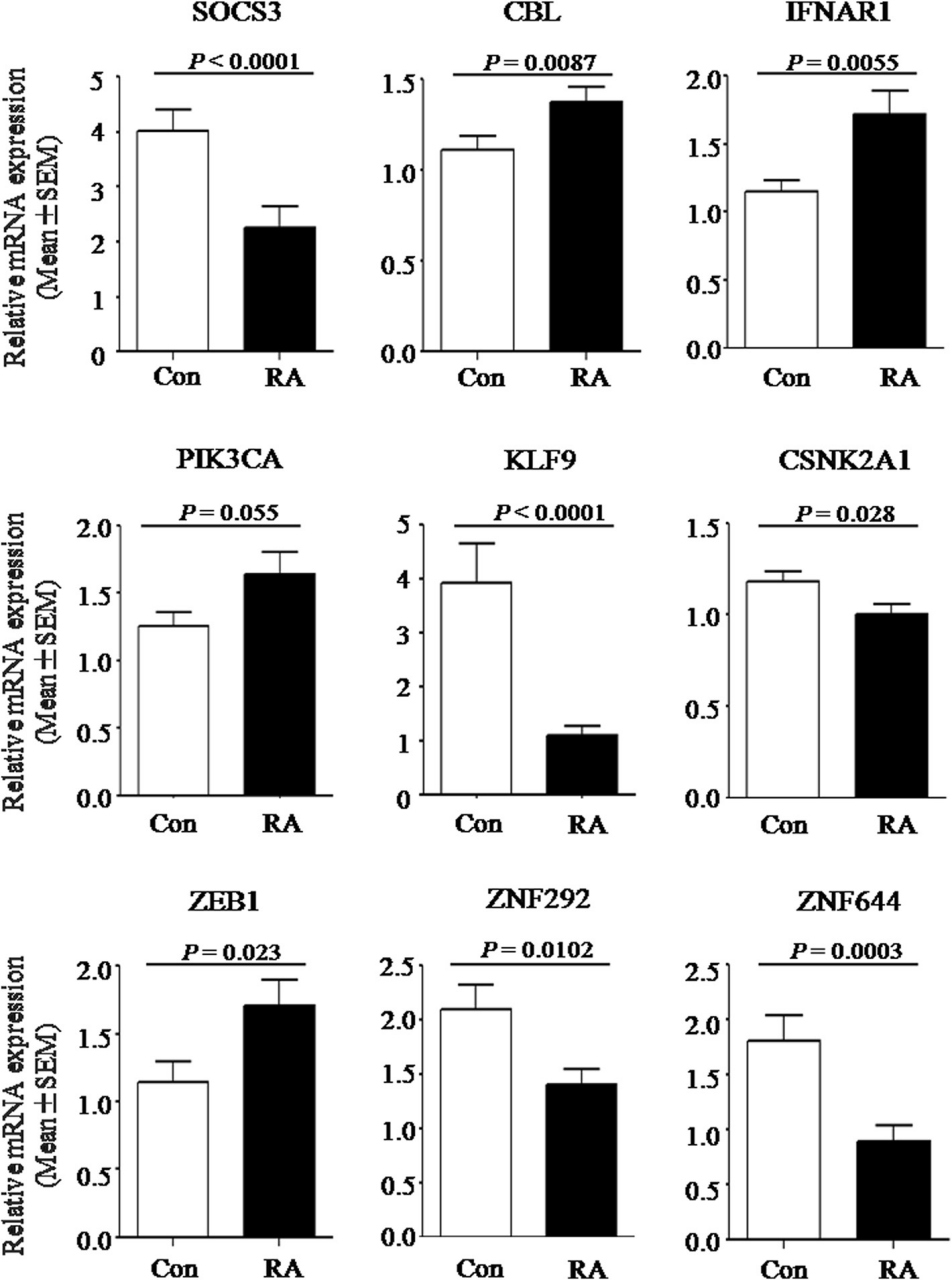
**Microarray results of gene expression profiles in CD4 T cells were validated by quantitative real-time polymerase chain reaction.** Independent-samples *t* tests were performed to compare rheumatoid arthritis (RA) patients (n = 40) with healthy individuals (n = 35). Data were presented as mean ± standard error of the mean (SEM). *P* value of less than 0.05 was considered statistically significant. Con, control.

qPCR was used to validate the selected 23 candidate genes. As shown in Figure 3, a robust differential expression was confirmed for eight of the 23 genes. One gene, PIK3CA, exhibited similar expression patterns but did not reach the statistical significance (*P* = 0.055). Interestingly, four out of the nine genes (SOCS3, CBL, IFNAR1, and PIK3CA) were noted to have STAT3 signaling involvement, and three genes (CBL, KLF9, and CSNK2A1) were involved in Wnt signaling pathway, based on the KEGG database and recent publications [25,26]. Additionally, several zinc finger transcription factors (ZEB1, ZNF292, and ZNF644) were confirmed.

## Discussion

RA is a chronic autoimmune disorder in which T cells play a pivotal role in the initiation and progression of the disease. Of these, CD4 T cells are key players in RA pathogenesis. A previous microarray study was conducted on CD4 T cells during the early phase of RA in a case-only design [10]. However, to date, no studies have focused on the expression profile of CD4 T cells between patients with RA and healthy individuals. Herein, we performed a unique CD4 T-cell transcriptome analysis in a case–control cohort. For the first time, we showed that CD4 T cells from patients with active RA and those from healthy controls had distinct gene regulations.

Our data indicate that the DE genes between two groups mainly involved in regulation of immune responses, especially T-cell response, and other cellular processes such as kinase and phosphatase regulator activity. There were also a significant number of genes involved in regulation of apoptosis. Apoptosis is a key process regulating immune homeostasis. Abnormalities in T-cell apoptosis resulted in a wide range of pathologic conditions. For instance, infection of CD4 T cells with HIV results in depletion of these lymphocytes because of programmed cell death [27]. In RA, one of the principal characteristics is ‘apoptosis resistance’ in the synovial tissues [28]. The synovial fluid T cells from the patients with RA were resistant to FasL-induced apoptosis [29]. Furthermore, accumulated evidence suggested that the Wnt signaling pathway plays an important role in FLS activation, bone resorption, and joint destruction in RA pathogenesis [30-34]. In the context of Wnt signaling in T-cell activation, it has been reported that activation of Wnt signaling in CD4^+^CD25^+^ regulatory T (Treg) cells increased the survival of Treg cells and induced an anergic phenotype in CD4^+^CD25^−^ effector T cells [35,36]. However, so far, few studies have focused on the involvement of Wnt signaling in T cells in RA development. Lee *et al*. [37] reported that sFRP1 (secreted frizzled-related protein 1), an inhibitor of Wnt signaling, was correlated with interleukin-17 (IL-17) levels in RA synovial fluid. *In vitro*, overexpression of sFRP1 induced a significant increase of T helper 17 (Th17) cells. In the present study, we observed that one of the significant GO categories was Wnt receptor signaling. Pathway analysis also indicates that Wnt signaling was one of the most significant pathways in the regulation of RA activity. Our findings support the notion that the Wnt signaling pathway may participate in the impaired T-cell homeostasis in RA. Notably, the genes involved in the SLE signaling pathway were also enriched in the present study, indicating that some of the genes are commonly shared by multiple autoimmune diseases, such as RA and SLE.

To reduce the complexity of the whole-genome expression data and to determine the most significant DE genes, we performed the gene module analysis to combine the results from GO, gene-gene interaction, and pathway analysis. In total, 23 genes were selected for further validation using a cut-off *q* value of 0.05 and an exclusion of being extensively studied in RA according to publications. Nine of those genes were confirmed in the validation cohort. Given the prominent importance of the STAT3 signaling pathway in many cellular functions, including T-cell differentiation [38], it is notable that four of the nine DE genes (SOCS3, CBL, IFNAR1, and PIK3CA) were involved in STAT3 signaling. SOCS3 is a key negative regulator that inhibits the STAT3 signaling pathway and is a major negative regulator of CD4 T-cell activation [39]. Huang *et al.* [40] reported that the expression of SOCS3 was elevated in patients with hepatitis C virus. The activation of SOCS3 contributes to the defective hepatic response to IFNγ. By contrast, reduced expression of SOCS3 has been observed in various human inflammatory diseases. Mice lacking *Socs3* developed an exacerbated inflammatory arthritis and were characterized by increased numbers of Th17 cells [41]. In the present work, SOCS3 expression was downregulated in RA CD4 T cells. The finding was consistent with a previous observation of the involvement of STAT3 signaling in patients with early arthritis [10]. Another interesting observation is that three validated transcripts (CBL, KLF9, and CSNK2A1) were dominantly related to Wnt signaling pathways. CBL is originally identified as a RING finger ubiquitin E3 ligase and recently has emerged as a key regulator of Wnt signaling by targeting the active β-catenin [25]. KLF9 is a transcriptional regulator of cell proliferation, adhesion, and differentiation and recently was shown to be a negative regulator of putative Wnt inhibitor DKK1 promoter activity in human stromal cells [26]. Furthermore, KLF9 was shown to function as a suppressor of tumor-initiating stem cells by directly suppressing Notch1 signaling [42]. As compelling evidence supports Notch involvement in CD4 T-cell differentiation [43-46], we speculate that KLF9 may function as a suppressor of CD4 T cells via the Notch signaling pathway and play a role in RA pathogenesis. Further functional study of the DE gene is needed to fully understand its contribution to RA. In addition, three zinc finger transcription factors (ZEB1, ZNF292, and ZNF644) were confirmed. Zinc finger proteins are the most abundant proteins in eukaryotes and play an important role in various cellular processes. Their functions are extremely diverse, including DNA recognition, apoptosis regulation, and transcriptional activation. Wang *et al.* [47] reported that ZEB1 acts as a specific repressor of IL-2 gene transcription and functions. Overexpression of ZEB1 can repress IL-2 promoter activity and endogenous IL-2 production in T cells. However, little is known about the role of the three zinc finger transcription factors in autoimmune diseases. Our results warrant the functional characterization of these zinc finger molecules to fully understand their contribution to RA pathogenesis.

## Conclusions

We report here the first case–control study of CD4 T-cell transcriptome profile in RA. Our data provide evidence that CD4 T cells from patients with RA had abnormal functional networks in the STAT3 signaling pathway and Wnt signaling. Our results also suggest that the aberrant expression of several zinc finger transcription factors (ZEB1, ZNF292, and ZNF644) may be potential pathogenic factors for RA.

## Additional files

Additional file 1: Table S1. Demographic characteristic of the healthy controls and patients with rheumatoid arthritis (RA) in study cohorts. Additional file 2: Table S2. Primer sequences for real-time quantitative polymerase chain reaction (PCR). Additional file 3: Table S3. A table listing the 832 upregulated genes in rheumatoid arthritis (RA) patients versus healthy controls. Additional file 4: Table S4. A table listing the 664 downregulated genes in rheumatoid arthritis (RA) patients versus healthy controls. Additional file 5: Table S5. A table listing the 122 differentially expressed (DE) genes involved in immune response. Additional file 6: Table S6. A table listing the 40 differentially expressed (DE) genes involved in T-cell response. Additional file 7: Table S7. A table listing the 111 differentially expressed (DE) genes involved in apoptosis process. Additional file 8: Table S8. A table listing the 89 differentially expressed (DE) genes involved in regulation of kinase and phosphatase activity. Additional file 9: Table S9. A table listing the 23 differentially expressed (DE) genes involved in regulation of Wnt receptor signaling. Additional file 10: Table S10. A table listing the gene modules involved in gene-gene interaction networks. Additional file 11: Table S11. A table listing the gene modules involved in pathway network analysis.

## Abbreviations

DE: differentially expressed
FDR: false-discovery rate
FLS: fibroblast-like synoviocyte
GEO: Gene Expression Omnibus
GO: Gene Ontology
HPRD: Human Protein Reference Database
IFNγ: interferon-gamma
IL: interleukin
KEGG: Kyoto Encyclopedia of Genes and Genomes
MINT: Molecular INTeraction
MPEA: metabolite pathway enrichment analysis
NCBI: National Center for Biotechnology Information
PBMC: peripheral blood mononuclear cell
qPCR: quantitative real-time polymerase chain reaction
RA: rheumatoid arthritis
sFRP1: secreted frizzled-related protein 1
SLE: systemic lupus erythematosus
STAT3: signal transducer and activator of transcription 3
TCR: T-cell receptor
Th: T helper
Treg: regulatory T
