# Supplementary material for: CD4 T-cell transcriptome analysis reveals aberrant regulation of STAT3 and Wnt signaling pathways in rheumatoid arthritis: evidence from a case–control study
Source: Arthritis Res Ther. 2015 Mar 22;17(1):76. doi: 10.1186/s13075-015-0590-9 (PMC4392874; doi:10.1186/s13075-015-0590-9)
Supplement: Additional file 1: Table S1. — Demographic characteristic of the healthy controls and patients with rheumatoid arthritis (RA) in study cohorts. [file 13075_2015_590_MOESM1_ESM.docx]

**Table S1** Demographic characteristic of the study cohorts

| **Characteristic** | **Discovery** | **validation** |
| --- | --- | --- |
| No. of patients | 13 | 40 |
| No. of controls | 9 | 35 |
| Female sex (patients, %) | 92.3 | 78.5 |
| Female sex (controls, %) | 100 | 62.8 |
| Age of patients (Mean ± SD years) | 50.3±7.2 | 56.85±12.89 |
| Age of controls (Mean ± SD years) | 46.7±11.9 | 39.15±9.8 |
| Disease duration (Mean ± SD years) | 8.7±7.2 | 7.12±6.5 |
| DAS 28 [median (IQR)] | 5.55 (4.07~9.26) | 7.31 (5.76~7.64) |

DAS 28: disease activity score; SD: standard deviation; IQR: Inter-Quartile Range.
