# Supplementary material for: CD4 T-cell transcriptome analysis reveals aberrant regulation of STAT3 and Wnt signaling pathways in rheumatoid arthritis: evidence from a case–control study
Source: Arthritis Res Ther. 2015 Mar 22;17(1):76. doi: 10.1186/s13075-015-0590-9 (PMC4392874; doi:10.1186/s13075-015-0590-9)
Supplement: Additional file 2: Table S2. — Primer sequences for real-time quantitative polymerase chain reaction (PCR). [file 13075_2015_590_MOESM2_ESM.docx]

**Table S2** Primer sequences for real-time quantitative PCR

| **No. Gene Forward primer Reverse primer Product**  **symbol length** | | | | |
| --- | --- | --- | --- | --- |
| 1 | CBL | GCAGTACAGGATCTCGTG | CTACCTCAACTCGGGAC | 107 |
| 2 | TRIM33 | GATCTGCTGAAGGTGACT | GAGGAGATTAACTGTGAGGGA | 100 |
| 3 | FAM91A1 | GGGTAATCATCTGATTCCGAG | CCAAATTCACAAAGCAAGGC | 119 |
| 4 | PRKCB | GAGAGACCATCAAGGGAAC | CAGGAGACTTTGCCTAAGC | 114 |
| 5 | MAP3K2 | GGATGTATGTTTGTGTGGAGA | ATCACAACTAAACAGTACCCT | 130 |
| 6 | ROCK1 | GAGACCTTCAAGCTCGAAT | AAGCATGTCTTGAGCCT | 104 |
| 7 | CSNK2A1 | GCAGTAACGGCCCTATC | CGGTGCTTCTGAAGTGT | 112 |
| 8 | ZAP70 | AGCCCTACAAGAAGATGAAAG | ATGAGTGCGTACAGTTCG | 103 |
| 9 | CCDC82 | AAGTAGGTGTTAAACGTCCC | TGAGAGTTCTTTGAGCTTCTG | 131 |
| 10 | ZNF292 | AGGTCACTGCTCATGTATAAC | TAGACTTACAGACTGCTGGT | 103 |
| 11 | PRKAA1 | CATGGATCTGTGTTCTGACTG | CTATTATGGGTGAGAAGATGAGG | 103 |
| 12 | PIK3R1 | GAAAGGCACGTCCACTCA | ACAAGTTAAACACTTTCTGGC | 102 |
| 13 | CSNK2B | CAGAGTGACCTGATTGAGC | GTACACACGAGGACAGTAAC | 138 |
| 14 | PPARD | AGCTCTCTTCCTGTCTTTG | AAGAAAGGAGGACAGACG | 155 |
| 15 | NOG | AGCGAGATCAAAGGGCTA | GACCACAGCCACATCTGTA | 101 |
| 16 | IFNAR1 | TTCCACATCACAGTATCTACCC | TGCAAATTCCAGCAGAAGCTA | 119 |
| 17 | PIK3CA | ACACTCAAAGAGTACCTTGT | GCAAATGGAAAGGCAAAGTC | 107 |
| 18 | SOCS3 | GAGACGGGACATCTTTCAC | GCATTTAAGGCGAATCTCTTAG | 100 |
| 19 | RPS6KB1 | CTGGAAGCCTTGGAATGG | CTGGTGTAAGAACAGGTGG | 103 |
| 20 | ZEB1 | TTCTCACACTCTGGGTCTTATT | GTGCTCATTCGAGAGGATTTC | 126 |
| 21 | ZNF644 | GCAGCATTTCAAAAGAACTGGCAC | CAACCACCACAGAGCTGACAAG | 107 |
| 22 | KLF9 | CTACAGTGGCTGTGGGAAAGTC | CTCGTCTGAGCGGGAGAACTTT | 129 |
| 23 | HNRNPA3 | TTATGGGTCGCGGAGGGAACTT | CTCCTCCATAACTACCTCTGCTG | 119 |
| 24 | 18S rRNA | CGGCTACCACATCCAAGGAA | GCTGGAATTACCGCGGCT | 187 |
